# Effects of Ti^4+^ Doping on the Structural Stability and Electrochemical Performance of Layered P2-Na_0.7_MnO_2.05_ Cathodes for Sodium-Ion Batteries

**DOI:** 10.3390/nano14241989

**Published:** 2024-12-12

**Authors:** Kexin Zheng, Jiawei Wang, Haifeng Wang, Zhengqing Pei, Qian Wang, Xinjie Zhou, Dehua Ma, Ju Lu

**Affiliations:** 1 School of Materials and Metallurgy, Guizhou University, Guiyang 550025, China; 18212076749@163.com (K.Z.); jwwang@gzu.edu.cn (J.W.); pzq13873253359@163.com (Z.P.); wang_qian0509@163.com (Q.W.); 18085758122@163.com (X.Z.); 18935592198@163.com (D.M.); 2642656691@163.com (J.L.); 2 Guizhou Provincial Key Laboratory of Metallurgical Engineering and Energy Saving, Guiyang 550025, China

**Keywords:** manganese base sodium-ion battery, Ti^4+^ doping, liquid-phase doping

## Abstract

The P2-Na_0.7_MnO_2.05_ cathode material has long been constrained by phase transitions induced by the Jahn–Teller (J–T) effect during charge–discharge cycles, leading to suboptimal electrochemical performance. In this study, we employed a liquid phase co-precipitation method to incorporate Ti during the precursor Mn_3_O_4_ synthesis, followed by calcination to obtain Na_0.7_Ti_x_Mn_(1−x)_O_2.05_ materials. We investigated the effects of Ti doping on the structure, morphology, Mn^3+^ concentration, and Na^+^ diffusion coefficients of Na_0.7_Ti_x_Mn_(1−x)_O_2.05_. Our findings revealed that the 7% Ti-doped NTMO-007 sample exhibited reduced grain agglomeration and smaller particle sizes compared to the undoped sample, thereby enhancing the electrode–electrolyte contact area and electrochemical activity. Additionally, Ti doping increased the crystal cell volume of Na_0.7_MnO_2.05_ and broadened the Na^+^ transport channels, significantly enhancing the Na^+^ diffusion coefficient. At a 0.5 C rate, the NTMO-007 sample demonstrated a specific capacity of 143.3 mAh g^−1^ with an 81.8% capacity retention after 100 cycles, markedly outperforming the undoped NMO sample, which had a capacity retention of only 61.5%.

## 1. Introduction

Lithium-ion batteries (LIBs) have become the dominant products in the field of electrochemical energy storage, finding extensive applications in new energy vehicles, artificial intelligence, and military sectors [1,2]. However, the limited reserves, challenging extraction processes, and high costs of lithium resources are increasingly constraining the widespread adoption of LIBs [3]. As portable electronic devices have proliferated and the new energy vehicle industry has developed rapidly in recent years, a significant amount of interest has been generated around sodium-ion batteries (SIBs) due to the abundance of sodium reserves. Unlike LIBs, which are heavily dependent on lithium resources, sodium is plentiful and widely distributed on Earth, conferring SIBs a natural advantage in raw material acquisition. Additionally, SIBs are considered green, safe, environmentally friendly, and sustainable, making them the best and most promising complementary product to LIBs [4,5,6]. A layered manganese-based transition metal oxide (Na_x_MnO_2_, x > 5) stands out as an exemplary cathode material for SIBs from the wide range of materials available [7,8]. The selection of manganese-based materials is due to their abundant availability, relatively favorable cost, excellent safety performance, and environmental friendliness [9,10].

The P2-type Na_0.7_MnO_2+y_ (0.05 ≤ y ≤ 0.25) cathode material has garnered extensive attention due to its high theoretical capacity and straightforward synthesis methods [11,12]. However, the structural integrity of the P2-type Na_0.7_MnO_2+y_ (0.05 ≤ y ≤ 0.25) material is compromised during prolonged cycling, as the Mn^3+^ (2Mn^3+^ → Mn^2+^ + Mn^4+^) undergoes disproportionation reactions [13]. The sustained dissolution of Mn^2+^ into the electrolyte leads to capacity degradation. Additionally, the Mn^3+^ ions are susceptible to the Jahn-Teller distortion, causing lattice distortion of the material and phase transition [14,15], which impedes Na^+^ diffusion rates. Therefore, the quest for developing layered manganese-based cathode materials that boast high capacities, superior performance, and low costs is of paramount importance for the advancement and proliferation of sodium-ion battery technology.

Researchers have utilized strategies such as metal doping, substitution, and coating to enhance materials’ electrochemical performance to address the aforementioned challenges [16,17,18]. Among these, metal ion doping involves the incorporation of foreign metal ions (e.g., Li^+^, Cu^2+^, Fe^3+^, Ti^4+^) into the crystal structure of Na_x_MnO_2_, either by replacing Na or Mn sites. Through this approach, it is feasible to mitigate Jahn–Teller distortions and enhance cycling stability and rate performance by optimizing the interlayer spacing required for Na^+^ diffusion, particularly in improving its rate capability. Consequently, a layer of manganese-based sodium-ion battery cathode material with metal ion doping is, therefore, a promising approach for fabricated high-performance cathodes. For instance, Rong et al. [19] proposed a method involving the substitution of Mn with a small amount of Li, which not only increased the reversible specific capacity but also effectively suppressed the phase transition. Similarly, Wang et al. [20] prepared Na_0.7_Mn_0.9_Cu_0.1_O_2_ through copper doping, demonstrating that Cu^2+^ inhibited the Jahn–Teller effect in the samples, resulting in superior storage performance compared to Na_0.7_MnO_2_. Similarly, the application of an AlPO_4_ coating to Na_0.7_MnO_2.05_ [21], serving as the cathode material in sodium-ion batteries, unveils notably enhanced electrochemical performance when compared to its uncoated counterpart. Lu et al. [22] employed polypyrrole (PPy) coating to modify Na_0.7_MnO_2.05_, enhancing the electrochemical performance and structural stability of the material, attributed to the PPy encapsulation. Among various metal ion dopants, Ti^4+^ (ionic radius: 0.605 Å) has garnered significant attention due to its close resemblance in ionic radius to Mn^4+^ (0.53 Å). This similarity allows for uniform incorporation of Ti^4+^ into Mn sites, making it a widely studied dopant. For example, Darbar et al. [23] implemented a titanium doping strategy to replace certain Mn and Fe atoms in P2-type Na_0.67_Fe_0.5_Mn_0.5_O_2_, thereby improving structural stability and electrochemical performance while mitigating the Jahn–Teller distortions caused by Mn^3+^ and Fe^4+^. Additionally, Tao et al. [24] incorporated Ti^4+^ into Na_0.67_Ni_0.33_Mn_0.67_O_2_, disrupting Na^+^ vacancy order and inhibiting irreversible multiphase transitions during Na^+^ deintercalation, leading to enhanced rate performance.

In this study, we propose a Ti^4+^ doping strategy to address the issue of poor electrochemical performance in P2-Na_0.7_MnO_2.05_, primarily due to Jahn–Teller distortions and P2-O2 phase transitions. By attempting to incorporate foreign metal ions and altering the local chemical environment within the crystal lattice, the goal is to achieve structural stability. We employed a liquid-phase co-precipitation method to incorporate Ti^4+^ into the precursor Mn_3_O_4_ during its synthesis, followed by high-temperature calcination to produce Na_0.7_Ti_x_Mn_(1−x)_O_2.05_ (x = 0, 0.03, 0.05, 0.07, 0.09) cathode materials. In this study, the effect of Ti element doping on the morphology, crystal structure, Mn^3+^ content, and cycling stability and rate performance of the samples was compared. The results indicate that the NTMO-007 sample, doped with 7% Ti, exhibits a high specific capacity of 143.3 mAh g^−1^ at a rate of 0.5 C. It retains 81.8% of its capacity after 100 cycles, significantly outperforming the undoped NMO sample (which retains 61.5%). This study enhances our understanding of how Ti doping affects the cycling and rate performance of P2-Na_0.7_MnO_2.05_, offering insights into the potential for further research on layered sodium manganate cathodes.

## 2. Experimental Section

### 2.1. Materials Synthesis

The synthesis of Na_0.7_Ti_x_Mn_1−x_O_2.05_ involves the following steps: Initially, Ti(SO_4_)_2_ (AR, Aladdin, Shanghai, China) and MnSO_4_·H_2_O (AR, Aladdin, Shanghai, China) are dissolved in 2 L of deionized water at a molar ratio of Mn:Ti = 1−x:x, with x ranging from 0 to 0.09 (x = 0, 0.03, 0.05, 0.07, 0.09). The resulting mixture is thoroughly homogenized. In a reactor, 600 mL of deionized water is added, and the system is stirred at a rate of 300 rpm. The temperature is elevated to 65 °C, after which O_2_ and NH_3_·H_2_O (AR, Aladdin, Shanghai, China) are simultaneously introduced until the pH reaches approximately 9. The addition rate of NH_3_·H_2_O is carefully regulated to maintain the pH around 9 throughout the 15 h reaction. Upon completion, the precipitate is allowed to cool naturally, filtered, and washed. It is then dried in an air atmosphere at 80 °C for 12 h, yielding Ti-doped Mn_3_O_4_ precursor. Subsequently, this precursor is mixed with Na_2_CO_3_ at a molar ratio of n(Na)/n(Mn) = 0.7:1 and thoroughly ground. The blend is calcined in a muffle furnace at 900 ℃ for 13 h, followed by cooling in the furnace. The resulting product is immediately vacuum-sealed and stored in an argon-filled glove box to prevent contamination from humid air. This process yields a series of Na_0.7_Ti_x_Mn_1−x_O_2.05_ materials (x = 0, 0.03, 0.05, 0.07, 0.09), which are designated as NMO, NTMO-003, NTMO-005, NTMO-007, and NTMO-009, respectively.

### 2.2. Material Characterization

The phases and structural properties of the samples were investigated using an X-ray diffraction (XRD) instrument (Bruker D8 Advance, Karlsruhe, Germany), employing a Cu Kα radiation source. The diffraction patterns were recorded at a scanning rate of 3° min^−1^. Particle morphology was analyzed using scanning electron microscopy (SEM, SU8020, HITACHI, Tokyo, Japan) and high-resolution transmission electron microscopy (HR TEM, FEI Tecnai G2 F30, 300 kV, FEI, Hillsboro, OR, USA). Elemental and valence compositions were determined via XPS (Thermo Escalab 250Xi, Thermo Fisher Scientific Inc., Waltham, MA, USA).

### 2.3. Electrochemical Testing

To fabricate the cathode, a homogenous blend of Na_0.7_Ti_x_Mn_(1−x)_O_2.05_, acetylene black, and polyvinylidene fluoride at a mass ratio of 8:1:1 was initially prepared. This mixture was then thoroughly ground with N-methyl-2-pyrrolidone (NMP) for a duration of 30 min to yield a uniformly dispersed slurry. The slurry was subsequently coated onto an aluminum foil and dried in a vacuum oven at 100 °C for 12 h. Post-drying, the coated aluminum foil was cut into circular cathode electrodes with a diameter of 12 mm. Each electrode retained an active material mass loading of approximately 2.08 mg/cm^2^. Utilizing sodium metal as the anode, a non-aqueous electrolyte consisting of 1 mol/L NaPF6, and a glass fiber separator with a diameter of 16 mm, we assembled a CR2032-type half-cell. These cells were tested on a battery test system (CT-4008T-5V) produced by Neware Electronics Co., Ltd. (Shenzhen, China), with galvanostatic charge–discharge tests and galvanostatic intermittent titration technique (GITT) conducted within a voltage range of 1.8–4.2 V. For GITT measurements, repeated current pulses of 0.1 C (where 1 C = 175 mAh g^−1^) were applied for 20 min, followed by a 120 min rest period. Electrochemical impedance spectroscopy (EIS) and cyclic voltammetry (CV) experiments were performed using a Donghua Analytical Electrochemical Workstation (DH7000). EIS tests spanned a frequency range of 100 kHz to 0.01 Hz with a 5 mV amplitude. CV measurements were conducted at a scan rate of 0.01 mV s^−1^ within the 1.8–4.2 V range.

## 3. Results and Discussion

**Figure 1:**
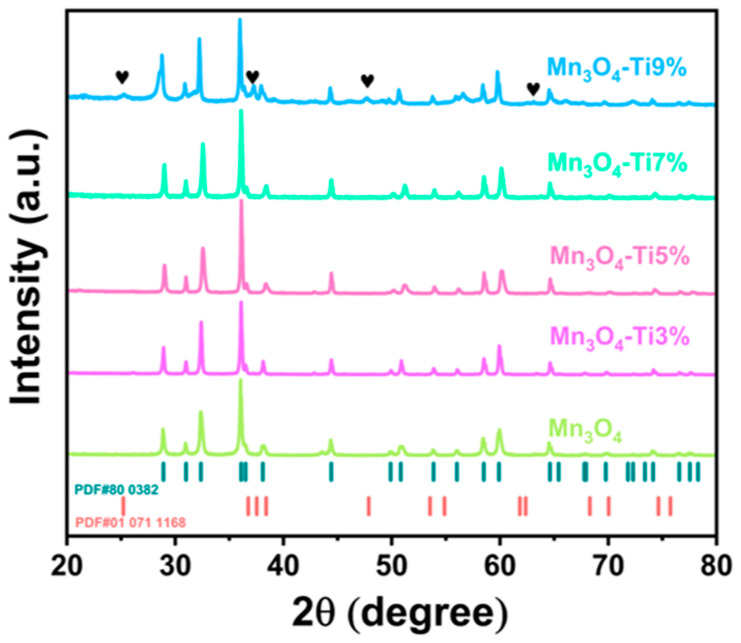
XRD patterns of precursor materials.

Figure 1 presents the X-ray diffraction (XRD) patterns of Mn_3_O_4_ precursors synthesized via coprecipitation, each exhibiting varying degrees of Ti doping. The diffraction peaks of each sample align well with the standard Mn_3_O_4_ card, characterized by flat baselines and sharp peaks, indicating that Ti doping does not alter the Mn_3_O_4_ crystal structure. Notably, in the Mn_3_O_4_-Ti9% spectrum, minor additional peaks not corresponding to Mn_3_O_4_ are observed, marked with “♥”. These peaks are attributed to titanium oxides, a consequence of excessive Ti content that remains unincorporated into the Mn_3_O_4_ lattice, coexisting as titanium oxides.

**Figure 2:**
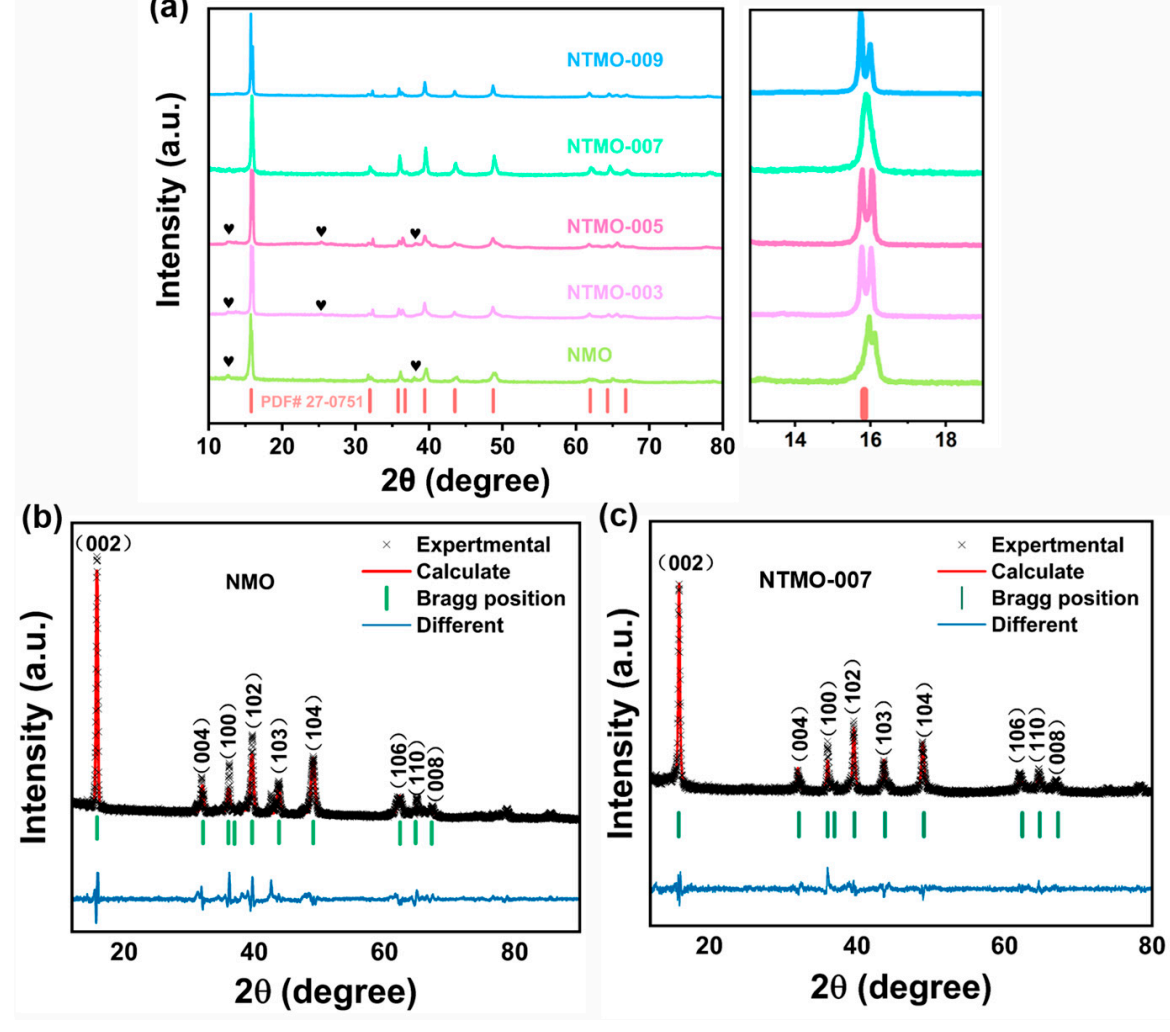
(**a**) XRD patterns of NMO, NTMO-003, NTMO-005, NTMO-007, and NTMO-009. (**b**) Rietveld refined XRD pattern of NMO. (**c**) Rietveld refined XRD pattern of NTMO-007.

To investigate the impact of Ti doping on the crystalline structure of the materials, X-ray diffraction (XRD) analysis was conducted on the NMO, NTMO-003, NTMO-005, NTMO-007, and NTMO-009 samples. Figure 2a presents the XRD patterns of the prepared samples in comparison with the standard spectrum of Na_0.7_MnO_2.05_. It revealed that all five samples have layered structures of a hexagonal P2-type [25,26], with a space group of P63/mmc (PDF: 27–0751). Upon closer inspection, minor extraneous peaks near 13°, 25°, and 38° were identified in the diffraction patterns of NMO, NTMO-003, and NTMO-005, marked with “♥”, which correspond to the characteristic diffraction peaks of NaMnO_2_ [27].

A comparative analysis of the (002) peaks reveals that the NTMO-003, NTMO-005, and NTMO-009 samples exhibit significantly broader (002) peaks with noticeable splitting compared to the NMO sample. This broadening is indicative of lower crystallinity. In contrast, the NTMO-007 sample exhibits a narrow and sharp (002) peak without any splitting, signifying superior crystallinity. To obtain deeper insights into the crystal structures, XRD Rietveld refinements of NMO and NTMO-007 samples were performed using GSAS-II [28], as illustrated in Figure 2b,c, with detailed results presented in Table 1. Comparative analysis of the cell parameters and volumes of the two samples reveals that the incorporation of Ti^4+^ leads to an expansion in the cell volume. This phenomenon arises because the ionic radius of Ti^4+^ is greater than that of Mn^4+^. Additionally, the doping of Ti elements leads to lattice expansion, resulting in an increase in both the lattice constants a and c. Notably, the c-axis, which represents the interlayer spacing of the TMO_2_ layers [29,30], grows larger, thereby increasing the interlayer distance, which is conducive to the extraction and insertion of Na^+^ ions, enhancing Na^+^ diffusion.

**Figure 3:**
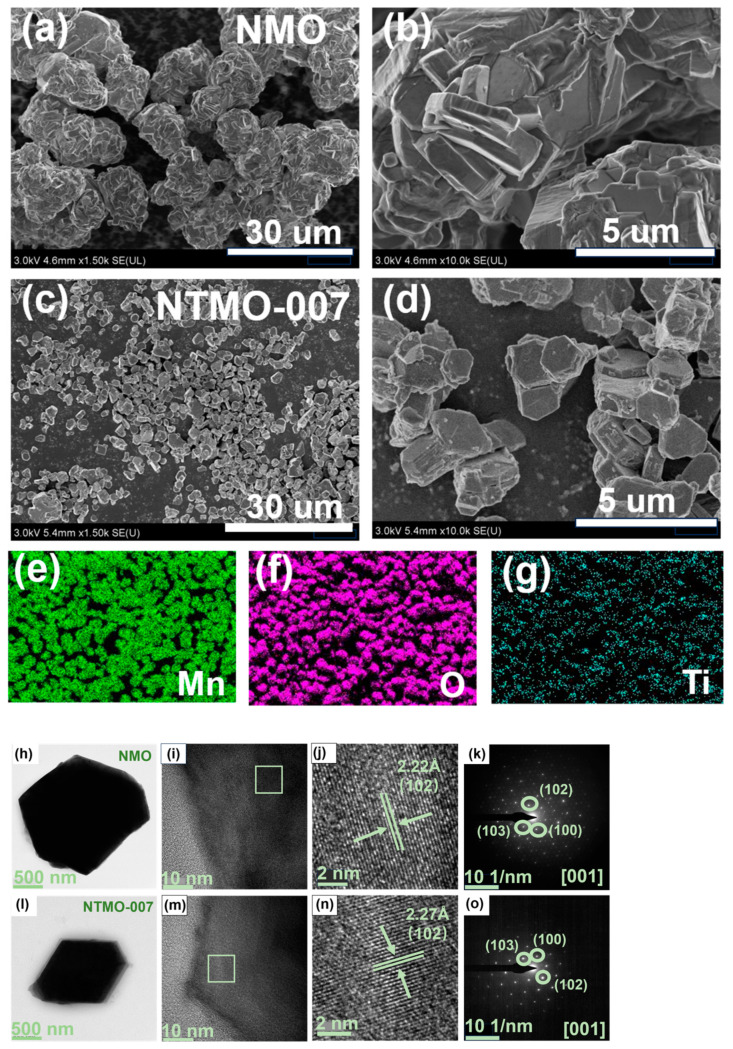
(**a,b**) SEM images of NMO, (**c,d**) SEM images of NTMO-007, (**e**–**g**) EDS images of NTMO-007, (**h**–**j**) HRTEM images of NMO, (**k**) SAED pattern of the NMO, (**l**–**n**) HRTEM images of NTMO-007, (**o**) SAED pattern of the NTMO-007.

The SEM images of NMO and NTMO-007 samples are presented in Figure 3a–d. The NMO sample appears as agglomerated spherical particles with irregular morphologies (Figure 3a,b). In contrast, the NTMO-007 sample consists of uniformly sized block-like particles, approximately 2 μm in diameter (Figure 3c,d). The elemental distribution of the P2-NTMO-007 particles, as shown in Figure 3e–g, reveals a homogeneous dispersion of Mn, O, and Ti across the sample. HRTEM images of NMO and NTMO-007 samples display distinct and consistent lattice fringes associated with the (102) crystal plane, demonstrating the materials’ high crystallinity. The distance between the lattice fringes is determined to be 2.22 Å and 2.27 Å, respectively. Based on the findings from XRD refinement, it is inferred that the enlarged lattice fringe spacing in NTMO-007 is a direct consequence of the larger Ti^4+^ ions incorporated within the TMO_2_ layer. A comprehensive analysis of the crystal structure was performed using SAED [31]. Figure 3k,o display the SAED images of the NMO and NTMO-007 samples, both of which can be indexed to the [001] zone axis. The corresponding crystal planes are (100), (102), and (103) of the P2 phase. There is no difference in exposed crystal planes between the two samples, demonstrating that Ti doping had no effect on the material’s original crystal structure.

**Figure 4:**
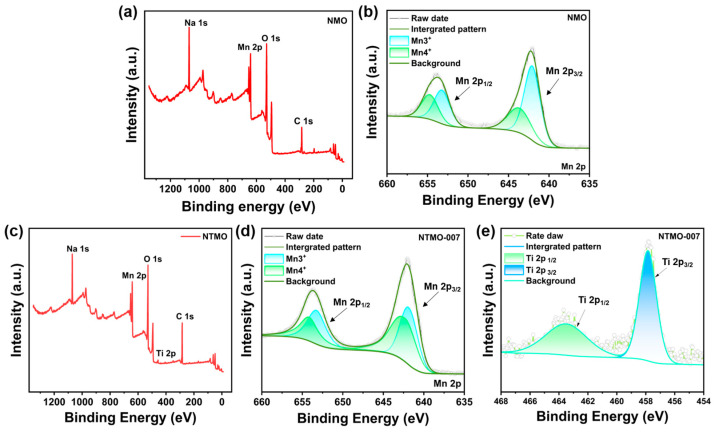
(**a**) XPS survey spectrum of the NMO sample, (**b**) XPS Mn 2p spectrum of the NMO sample, (**c**) XPS survey spectrum of the NTMO-007 sample, (**d**) XPS Mn 2p spectrum of the NTMO-007 sample, (**e**) XPS Ti 2p spectrum of the NTMO-007 sample.

The XPS analysis was performed on the samples to determine their chemical composition and valence state. In Figure 4, we show the resulting total XPS spectra and fitted curves for NMO and NTMO-007 samples. A comparison of the survey spectra of both samples is presented in Figure 4a,c. A comparison of the full spectra reveals that both NMO and NTMO-007 exhibit characteristic peaks for Na 1s, Mn 2p, O 1s, and C 1s, with no additional impurity peaks. After carbon correction, the XPS spectra were fitted and analyzed. The NTMO-007 full spectrum shows a characteristic peak for Ti 2p. In NTMO-007, Ti 2p peaks were observed at 457.8 and 463.6 eV, corresponding to Ti 2p3/2 and Ti 2p1/2, respectively (Figure 4e). The detailed fitting of these peaks indicates that Ti is in the +4 oxidation state within the material, confirming that Ti was successfully incorporated. Figure 4b,d, respectively, show the fitted Mn 2p fine spectra for NMO and NTMO-007 samples. Based on the XPS results for Mn 2p1/2 and Mn 2p3/2, both samples contain Mn^3+^ and Mn^4+^ [8]. The Mn 2p3/2 peaks for NMO and NTMO-007 are observed at 641.9 eV and 642.1 eV, respectively, while their Mn 2p1/2 peaks appear at 653.7 eV and 653.8 eV. Based on the peak areas of the Mn 2p features, it is possible to calculate the specific ratio of Mn^3+^ to Mn^4+^. The area ratio for Mn^3+^ to Mn^4+^ in the NMO sample is 1.74, whereas it is 0.99 in the NTMO-007 sample, indicating that the incorporation of Ti effectively reduces the number of Mn^3+^ ions and enhances the overall valence state of the Mn ions, thereby mitigating Jahn–Teller distortions caused by Mn^3+^. This enhancement contributes to the improved structural stability of the material.

**Figure 5:**
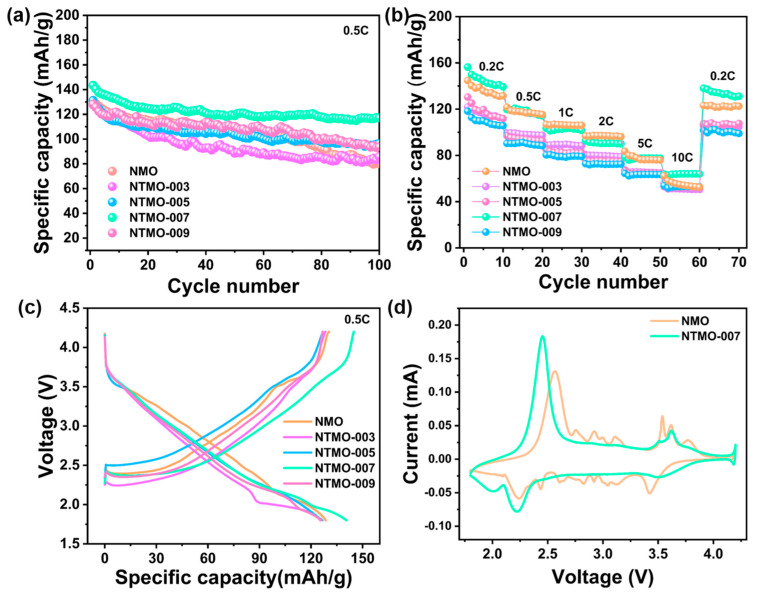
(**a**) Cyclic performance of NMO, NTMO-003, NTMO-005, NTMO-007, and NTMO-009 at 0.5C within the voltage range of 1.8–4.2 V, (**b**) rate performance of NMO, NTMO-003, NTMO-005, NTMO-007, and NTMO-009, (**c**) second cycle charge–discharge curves of NMO, NTMO-003, NTMO-005, NTMO-007, and NTMO-009, (**d**) cyclic voltammetry (CV) curves of NMO and NTMO-007 samples.

To evaluate the stability of Ti-doped samples, we conducted cycling and rate performance tests on NMO, NTMO-003, NTMO-005, NTMO-007, and NTMO-009. Within a voltage range of 1.8–4.2 V and at 0.5 C, the initial capacities of these five samples after 100 cycles were 130.3, 127.7, 131.3, 143.4, and 128.3 mAh g^−1^, respectively. The capacity retention rates after 100 cycles were 61.5%, 67.5%, 71.4%, 81.8%, and 73.1%. As the Ti doping level increased, the cycling stability of the samples first improved and then gradually declined. This trend can be attributed to the partial substitution of Mn^3+^ by Ti, which suppressed the Jahn–Teller effect and enhanced cycling stability. However, the decrease in cycling stability observed in the NTMO-009 sample may be due to the presence of titanium oxides coexisting with Mn_3_O_4_ in the precursor, as some Ti could not be doped into Mn_3_O_4_. This impurity may have affected the crystallinity of Na_0.7_MnO_2.05_ during subsequent calcination, thereby impacting the cycling performance of NTMO-009. As shown in Figure 5a, the NTMO-007 sample exhibited a high initial capacity and superior cycling stability after 100 cycles compared to the undoped NMO sample. We also compared the rate performance of samples with different Ti doping levels to that of NMO (Figure 5b). The NTMO-007 sample demonstrated rate capabilities of 156.4, 119.7, 103.4, 92.8, 78.4, and 63.8 mAh g^−1^ at 0.2 C, 0.5 C, 1 C, 2 C, 5 C, and 10 C, respectively. When the current was returned to 0.2 C, the capacity was still 138.1 mAh g^−1^. These results suggest that appropriate Ti doping can enhance the structural stability of the material, reduce phase transitions during charge–discharge processes, and thereby improve the cycling stability and rate performance of the samples [24,32].

The second cycle charge–discharge curves of the five samples, as illustrated in Figure 5c, reveal a progressive smoothness with increasing Ti doping. The undoped NMO sample exhibits a step-like pattern with multiple voltage plateaus, whereas NTMO-007 demonstrates a more streamlined profile. This suggests that moderate Ti incorporation into the P2-type structure effectively smoothens the charge–discharge curves, facilitating phase transition suppression and stabilizing the crystal structure during cycles. It is noteworthy that all Ti-doped samples display slightly reduced crossover potentials compared to NMO. This reduction is attributed to the rearrangement and migration of Na^+^ vacancies and the formation of intermediate phases within the mid-voltage range, leading to numerous minor voltage plateaus at mid-voltages, which delay the voltage decline in NMO near the mid-range and result in higher crossover potentials compared to the doped samples. Cyclic voltammetry (CV) tests were conducted to elucidate the electrochemical behaviors of the prepared samples. The CV curves of NMO and NTMO-007, depicted in Figure 5d, show that both samples exhibit sharp peaks around 2.2–2.6 V, corresponding to the reversible redox peaks of Mn^3+^/Mn^4+^. NMO exhibits a series of additional peaks around 2.5–4.0 V, while NTMO-007 maintains a smoother CV curve. This disparity arises from the rearrangement and migration of Na^+^ vacancies and intermediate phase formation occurring within the 2.6–4.0 V range during cycling in NMO, which negatively impacts long-term cycling stability. The smooth CV curve of NTMO-007 indicates that Ti incorporation effectively inhibits Na^+^/vacancy ordering and intermediate phase formation, enhancing cycling reversibility. Additionally, both samples exhibit additional peaks around 4.2 V, attributable to the high-voltage phase transition of the P2 phase due to excessive sodium removal at high potential [33].

**Figure 6:**
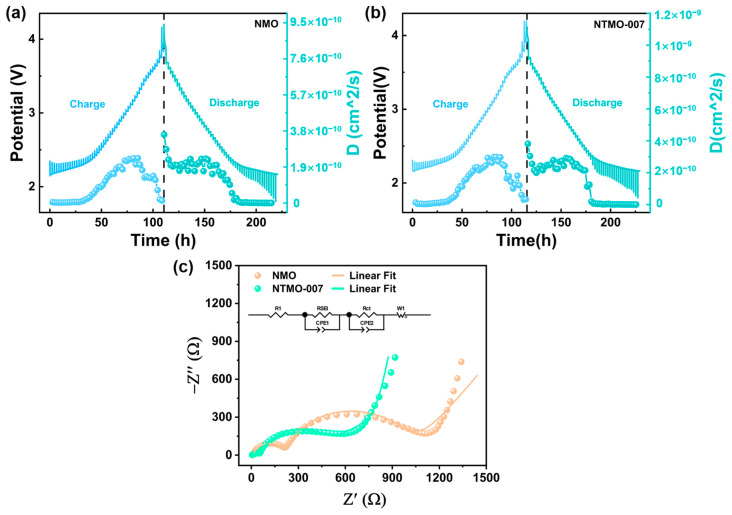
(**a**,**b**) GITT curves and the calculated diffusion coefficients during charging and discharging for the NMO and NTMO-007 samples, (**c**) Nyquist plots of NMO and NTMO-007 samples after 100 cycles.

We calculated the diffusion coefficients of Na^+^ using the GITT in order to determine the effects of Ti doping on sodium ion diffusion kinetics [33]. The GITT experimental results for NMO and NTMO-007 are, respectively, presented in Figure 6a,b. The data on their Na^+^ diffusion coefficients are listed in Table 2 Equation (1) was utilized to compute the diffusion coefficients of sodium ions.

(1) $$D_{{Na}^{+}}=\frac{4}{\Pi \tau }{\left(\frac{m_{B}V_{M}}{M_{B}S}\right)}^{2}\left(\frac{{\Delta E}_{s}}{{\Delta E}_{\tau }}\right))$$

where *τ* denotes the relaxation time, a mass is given by *m_B_* (g) and a molar volume is given by *V_M_* (cm^3^/mol), *M_B_* (g/mol) is the electrode material’s molar mass, while *S* (cm^2^) is the effective electrode–electrolyte contact area. A constant current pulse and steady-state (equilibrium) cell voltage E are represented by ΔE*_s_* and ΔE*_τ_*, respectively. As depicted in the figure, both NMO and NTMO-007 samples exhibit Na^+^ diffusion coefficients of approximately 10–10 cm^2^ s^−1^. However, the diffusion coefficient of NTMO-007 is noticeably higher, indicating that the doping of Ti significantly enhances the sluggish sodium ion diffusion kinetics caused by phase transitions, thereby increasing the diffusion rate of sodium ions [34,35].

To elucidate the Na^+^ transport kinetics of NMO and NTMO-007, we conducted electrochemical impedance spectroscopy (EIS) measurements on both samples. Figure 6c presents the Nyquist plots of NMO and NTMO-007 after 100 cycles of activation. Table 2 summarizes the corresponding values of R_SEI_ resistance and R_ct_. After cycling, both samples have a semicircle at high frequencies, a semicircle at mid frequencies, and a straight line at low frequencies on their Nyquist plots, corresponding to the R_ct_ of the interface, the SEI film resistance R_SEI_, and the Warburg impedance, respectively. As indicated in the table, after 100 cycles, the sum of R_ct_ and R_SEI_ for NTMO-007 is lower than that for NMO, demonstrating that the doping of Ti effectively enhances the stability of the interface and maintains good Na^+^ transport capability.

## 4. Conclusions

In summary, the incorporation of Ti into the Na_0.7_MnO_2.05_ cathode material results in a more dispersed crystalline morphology, thereby reducing the overall Mn^3+^ content and expanding the Na^+^ transport channels. This modification effectively mitigates capacity decay caused by the Jahn–Teller effect during charge–discharge cycles. Specifically, the NTMO-007 sample, following Ti doping, exhibits improved Na^+^ diffusion rates and significantly enhanced cycling and rate performance. Notably, it demonstrates a high specific capacity of 143.3 mAh g^−1^ at a rate of 0.5C, retaining 81.8% of its capacity after 100 cycles.

## Figures and Tables

**Table 1:** Cell parameters derived from Rietveld refinement for NMO and NTMO-007.

Samples	a(b) (Å)	c (Å)	V (Å)	R-Pattern	R_wp_	Chi^2^
NMO	2.871	11.148	V = 79.574	9.56%	11.63%	2.01
NTMO-007	2.893	11.155	V = 80.292	8.86%	10.43%	1.83

**Table 2:** Sodium ion diffusion coefficients, R_SEI_, and R_ct_ values for the NMO and NTMO-007 samples.

Samples	D_Na+_	R_SEI_ (Ω)	R_ct_ (Ω)	R_SEI_ + R_ct_ (Ω)
NMO	1.094E−10	175.1	654.7	829.8
NTMO-007	1.322E−10	53.6	366.2	419.8

## Data Availability

The data presented in this study are available on request from the corresponding author.
